# Improving health-seeking behaviours of older adults in urban Africa: A holistic approach and strategic initiatives

**DOI:** 10.7189/jogh.14.03009

**Published:** 2024-02-09

**Authors:** Daniel Katey, Abigail Agyekum, Anthony Kwame Morgan

**Affiliations:** 1 Department of Geography and Rural Development, Kwame Nkrumah University of Science and Technology, Kumasi, Ghana; 2 Trent Centre for Aging & Society, Trent University, Peterborough, Ontario, Canada; 3 Avery Healthcare, Christchurch Road, Virginia Water, UK; 4 Department of Planning, Kwame Nkrumah University of Science and Technology, Kumasi, Ghana

## BACKGROUND AND GENERAL OVERVIEW

The health-seeking behaviours of older adults (aged ≥60) in urban Africa remain a major concern as the continent undergoes rapid urbanisation and demographic changes. Generally, health-seeking behaviours are people’s activities to seek medical advice, diagnosis, and treatment when they believe they are ill or need health care services [1,2]. Health-seeking behaviours, in essence, show the proactive measures individuals take to address their health needs and well-being, enabling a holistic approach to live an active and meaningful life. These behaviours may include seeking expert medical counsel and therapy to adopt healthier lifestyles, including regular exercise and balanced dieting. Thus, the growing ageing population in African cities necessitates a better understanding of how older adults seek healthcare services and what variables impact their health-seeking behaviours [3]. Urbanisation in Africa has resulted in major demographic shifts, characterised by declining fertility rates and increasing life expectancy, with more older adults now residing in urban areas [4]. This change has created specific health challenges for older age groups, including changing lifestyles, reduced utilisation of health care services, and probable social isolation [3,4]. Moreover, Africa’s older adult population has been predicted to rise from 64 million in 2015 to about 105 million by 2030 [4]. Hence, understanding the health-seeking behaviours of older people on the continent is critical for developing focused healthcare strategies to address their specific needs and challenges.

Several factors have been identified as major influencers of the health-seeking behaviours of older adults in urban Africa. For instance, Govender et al. [5] and Nakovics et al. [6] identified high out-of-pocket expenditures for healthcare services and inadequate access to healthcare facilities among older adults in South Africa and Malawi. Health illiteracy and knowledge deficiencies are notable concerns within the population in Ghana [7]. Further evidence suggests that older adults may have a poor grasp of their health conditions and may be unaware of accessible healthcare options, which may result in delayed or inappropriate care-seeking behaviours [8]. More so, issues including cultural attitudes and lack of trust in formal medical settings have led to some older adults preferring traditional healers over contemporary healthcare providers [8]. In line with these challenges, some programmes have been implemented to promote health-seeking behaviours among the region's older adult population. These activities include community health campaigns, health education programmes, and the implementation of national health insurance schemes in some countries, including South Africa, Nigeria, Ghana, and Kenya [9-11].

Despite the plethora of existing programmes, it appears that some difficulties exist that could jeopardise the efficacy of these initiatives. For instance, while community health initiatives and health education programmes are important, reaching all older adults in urban settings might be challenging [12]. Additionally, some older adults may experience social isolation or encounter challenges in accessing information, thereby complicating awareness of health care services [13]. In some areas of urban Africa, health care infrastructure may still be deficient or inadequate, particularly in some disaster-burdened countries like Somalia, Burundi, Mali, the Central African Republic, Niger, Eritrea, and Togo [12,14,15]. Also, even when health insurance programmes are in place, the quality and accessibility of health care facilities may not satisfy the needs of older persons, forcing them to be hesitant to seek medical care [16]. Undoubtedly, these issues are major threats to the World Health Organization (WHO) healthy ageing initiative, which seeks to create the environments and opportunities that allow people to do what they value throughout their lives. Therefore, addressing the health-seeking behaviours of urban African older populations necessitates a holistic approach, considering the complex interplay of socio-spatial, cultural, political, economic, and infrastructure issues. Consequently, this study posits the following considerations to help improve the health-seeking behaviours of older adults in urban Africa.

## RECOMMENDATION

First and foremost, there is a need to prioritise health care accessibility and affordability. Thus, it is crucial to enhance access to medical services by both increasing availability and reducing costs, especially in highly impoverished countries including Angola, Mauritania, Ethiopia, South Sudan, the Democratic Republic of Congo, Mozambique, Sudan, and Madagascar. A recent study published in The Lancet Healthy Longevity identified these countries as having many densely populated areas with limited physical access to healthcare facilities for older adults [17]. Also, national governments should subsidise healthcare expenditures for older people and invest in urban healthcare infrastructure [17,18]. The subsidisation of some health expenditures for older adults appears to work in some advanced countries like the United Kingdom and Canada, where some older adults are entitled to free drugs and medical supplies, including allergy shots, needles, alcohol swabs, and glucose test strips. This helps to alleviate financial burdens related to some essential medications among older people. However, while these recommendations are consistent with the WHO’s objective of enhancing geriatric care, adopting such initiatives might be challenging, particularly in most of Africa's low- and middle-income countries. For instance, economic differences, the quality of healthcare infrastructure, and limited financial resources could pose significant threats to implementing a universal healthcare subsidisation programme. Also, differences in governance structures and policy frameworks among countries may limit the practicality of reproducing models, such as the free medical prescriptions for older people across all African countries. Nevertheless, tailoring these strategies to suit the specific needs, resources, and governance structures of individual African countries could be essential to enhance the feasibility and effectiveness of urban healthcare systems for older populations.

Second, there is a need for health education and awareness creation. Initiatives focusing on health education and raising awareness about prevalent health conditions affecting older persons can give them the knowledge they need to make well-informed choices about medical care [18]. Community convergences, the media, and local healthcare centres can all be leveraged to launch targeted campaigns. In doing this, local languages could be used as mediums of communication to ensure that information is easily understood and relatable to older adults who may not be fluent in official languages such as English and French. Also, community leaders, elders, and local organisations should be involved in the planning and implementation of health education programmes since their endorsement and active participation could enhance trust and engagement among older people [18]. However, given the culturally diverse nature of the African continent, some difficulties may likely develop in areas with a wide range of dialects and linguistic diversity, especially in sub-Saharan Africa. Anecdotal evidence suggests that West Africa has the most language-dialect diversity in the region, with Nigeria alone having over 500 dialects, followed by Cameroon, Ghana, and Senegal. Hence, to ensure that important health-related information is properly transmitted to specific groups of older adults, there is a need to consider these entrenched local sociocultural dynamics as well.

Third, early screening to detect potential health disorders or diseases and regular check-up incentives should be introduced. Encouragement of frequent health check-ups and screenings for older persons can benefit the early diagnosis of health conditions [19]. Offering incentives such as lower costs or health insurance benefits can encourage people to seek health care frequently, thereby making health care more accessible as well as encouraging people to seek treatment without fear of financial upshots. Nevertheless, due to economic variations and the low quality of most existing healthcare infrastructure in some countries [18], the viability of this proposition may differ across the continent. Hence, specific government initiatives, public-private partnerships, and community participation would be required. Also, addressing financial constraints and building a culture of preventive care will be critical to the success of such projects in urban Africa.

Finally, mobile clinics and health outreach programmes should be introduced into older adult healthcare systems in Africa. These can serve older adults with restricted mobility or difficulty accessing healthcare facilities. These programmes have the potential to offer medical care directly to their communities. So, the mobile clinics and outreach teams need to include interdisciplinary healthcare teams that comprise doctors, nurses, chemists, psychologists, and social workers. These multidisciplinary teams can provide comprehensive treatments while addressing key medical and social needs. Furthermore, mobile clinic timetables should be created to meet the special needs of older people while considering influences such as major market days, religious occasions, traditional festivals, and agricultural activities. However, if mobile clinics and outreach programmes become impracticable (probably due to socio-spatial and financial constraints), telemedicine and other technology-based approaches could be resorted to [20]. These may entail virtual consultations that could help overcome geographical and financial constraints and provide medical advice from a distance [20]. Yet, given the generally low level of formal education across the continent coupled with inaccessibility to mobile devices and mobile phone networks, this option should be implemented cautiously. Although mobile clinics can address mobility issues and enhance accessibility, potential barriers may include logistic and funding challenges and the need for skilled interdisciplinary healthcare teams. Overcoming these challenges would require strategic planning, collaboration with local communities, and adapting schedules to align with cultural events and activities. Additionally, incorporating telemedicine could be a viable alternative; however, its success may depend predominantly on overcoming barriers related to low levels of formal education and digital literacy among older adults as well as limited access to mobile devices and reliable telecommunication networks.

## CONCLUSIONS

Addressing the health-seeking behaviours of older adults in urban Africa demands a comprehensive approach encompassing improved healthcare accessibility and affordability, culturally sensitive health education, incentivised early detection measures, and the introduction of tailored mobile clinics and outreach programmes. Overcoming financial barriers, health illiteracy, and inadequate infrastructure is also pivotal in promoting informed and proactive healthcare decisions. While at it, ageism in healthcare accessibility needs to be mitigated since it can result in the neglect of tailored services, limited accessibility, and insufficient attention to the unique health requirements of elderly populations [21,22]. Downplaying the effects of ageism in healthcare delivery could lead to a failure to recognise and respond to the distinct healthcare needs of older individuals, thereby contributing to disparities in health outcomes and overall well-being. Ultimately, by embracing these strategies, urban African societies can pave the way for “healthy ageing”, eventually fostering a more inclusive and equitable healthcare landscape that values and supports the well-being of older adults.
